# H Syndrome retrospectively diagnosed: The importance of recognizing cutaneous signs

**DOI:** 10.1002/ccr3.3082

**Published:** 2020-07-14

**Authors:** Aurora Parodi, Emanuele Cozzani

**Affiliations:** ^1^ DiSSal Section of Dermatology San Martino Polyclinic Hospital University of Genoa Genoa Italy

**Keywords:** cutaneous hyperpigmentation, genodermatosis, H syndrome, hypertrichosis, induration

## Abstract

We present a case of a retrospectively diagnosed H syndrome in a man who died of a probable heart infarction. We highlight the importance of recognizing cutaneous hallmarks of this syndrome for better clinical management and prevention.

## Graphical abstract

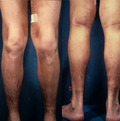

## 1 INTRODUCTION

H syndrome is a rare autosomal recessive genodermatosis with a possibly severe multisystem involvement. The major cutaneous signs are cutaneous hyperpigmentation, induration, and hypertrichosis which start on the legs but may become more widespread. These features should be considered as the pathognomonic clinical signs of H syndrome.

## 2 CASE HISTORY

**FIGURE 1:**
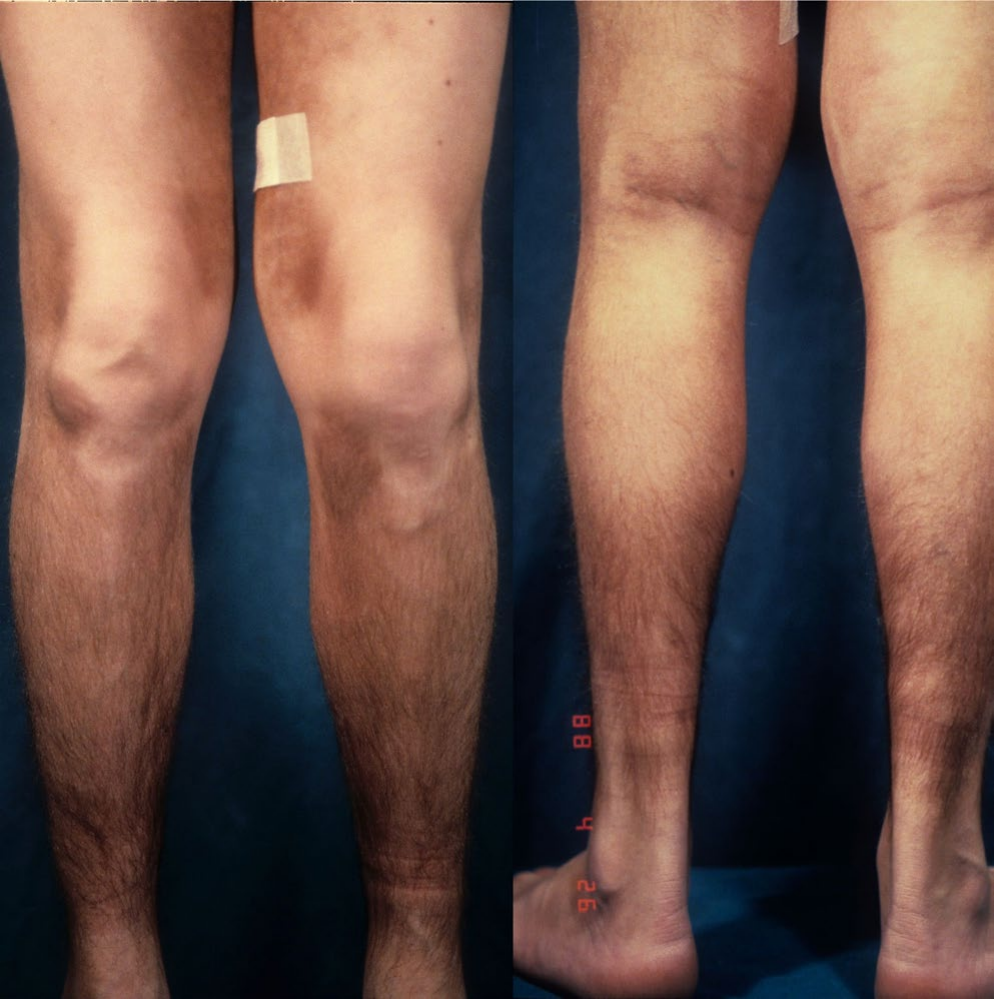
Indurated plaques with hypertrichosis of the legs

We present a case of H syndrome, diagnosed retrospectively, in a man who probably died of heart infarction. Recognizing cutaneous hallmarks of this syndrome is very important for a better clinical management and to prevent severe and possibly fatal complications, as occurred in our patient. In 1980, we visited an 18‐year‐old man who presented with brown‐black, patches of hypertrichosis on his inner thighs and legs (Figure 1). The lesions were indurated and asymptomatic and had appeared during his infancy. The patient also showed flexion contractures of the fingers, short stature, hypogonadism, and flat feet. In addition to this, he suffered of insulin‐dependent diabetes, diagnosed at the age of 7. Raynaud's phenomenon was not reported. The patient had learning difficulties probably due to a modest mental disability. No family diseases or comorbidities were reported.

## 3 DIFFERENTIAL DIAGNOSIS, INVESTIGATIONS, AND TREATMENT

In the hypothesis of an “atypical” morphea, atypical for the presence of hairs in the plaques, we performed a biopsy on one of the hyperpigmented plaques. The histopathologic findings revealed a slight acanthosis, increased melanin deposition in basal keratinocytes, and a perivascular mononuclear infiltrate in the superficial and mid dermis. A mild dermal fibrosis was present, but the adnexa were spared. Laboratory tests including antinuclear antibodies, rheumatoid factor, C‐reactive protein, and erythrocyte sedimentation rate were negative or within normal limits. The patient was discharged from our Institute with a diagnosis of morphea‐like lesions sparing follicular adnexa in an insulin‐dependent diabetic patient. Since our evaluation, the patient, followed for insulin‐dependent diabetes, continued being in apparent good health. At the age of 37, without systemic symptoms or particular clinical manifestations, the patient suddenly died, probably of heart infarction in another hospital. The autopsy was not performed because the relatives did not consent to it. Retrospectively, we were able to diagnose that the patient had H syndrome.

## 4 DISCUSSION

H syndrome is a rare syndrome, first described in 2010. Its name comes from the multiple Hs that represent the most frequent clinical features: hyperpigmentation, hypertrichosis, hepatosplenomegaly, hearing loss, heart abnormalities, hypogonadism, low height (short stature), hyperglicemia (diabetes mellitus), and hallux valgus (and flexion contractures).^1^, ^2^, ^3^, ^4^ The majority of patients with H syndrome are of Arabic origin, although patients from various parts of the world have been described.^2^ Our patient was Caucasian from Southern Italy. The most common clinical finding is cutaneous hyperpigmentation, together with hypertrichosis and induration, observed in 68% of patients with an average age of onset of 9.7 years. Flexion contractures of the fingers and toes are the second most common feature (56%). Sensorineural hearing loss is the third most common clinical finding (53%). Short stature is seen in 49%, hepatomegaly in 43%, and splenomegaly in 39%. Cardiac anomalies, hallux valgus, genital masses, dilated lateral scleral vessels or episcleritis, exophthalmos or eyelid swelling, varicose veins, and facial telangiectasia, in decreasing order, are present in a third to a quarter of patients. Numerous cardiac abnormalities are described, the most common of which are pericardial abnormalities. Lymphadenopathy which may be generalized or localized, with inguinal, cervical, and axillary nodes most commonly affected, is present in 24% of patients, mimicking a Rosai‐Dorfman disease. Insulin‐dependent diabetes mellitus was observed in 23% of patients. Hypogonadism was reported in 16% of cases.^2^ The histopathologic findings include widespread fibrosis; striking mononuclear infiltrates consisting mainly of monocyte‐derived cells (small CD68 histiocytes and CD34 and FXIIIa dendrocytes) and plasma cells; thickened, fragmented, and partially calcified elastic fibers, admixed with well‐formed psammoma bodies, a previously unrecognized feature in non‐neoplastic skin and subcutaneous conditions. In addition, the ultrastructure of CD68 small histiocytes exhibits distended endoplasmic reticulum and scarcity of lysosomes, features typical for fibroblasts but unusual for histiocytes.^5^ H syndrome is an autosomal recessive genodermatosis with multisystem involvement caused by increasing of the spectrum of known SLC29A3 mutations.^6^, ^7^ The hallmarks of the syndrome are cutaneous hyperpigmentation, induration, and hypertrichosis, which initially start on the legs and may later become more widespread. These features should be considered as the pathognomonic clinical signs of H syndrome. Unfortunately, in our case, the molecular analyses were not performed. Nevertheless, it is important to highlight the importance of the cutaneous signs of this syndrome. We recommend clinicians to take into consideration the diagnosis of this rare H syndrome, when finding these cutaneous features, to prevent severe complications, as occurred in our patient. Finally, it is important to emphasize that the syndrome has a wide clinical variability and that patients can be oligosymptomatic (eg, only IDDM). Therefore, clinicians should maintain a high index of suspicion and all family members should be evaluated, even if there are only mild or no symptoms.

## CONFLICT OF INTEREST

None declared.

## AUTHOR CONTRIBUTIONS

AP: involved in ideation, data analysis, writing, revision, and final approval. EC: involved in data analysis, writing, revision, and final approval.
